# Supplementary material for: Generation, transmission, and conversion of orbital torque by an antiferromagnetic insulator
Source: Nat Commun. 2025 Oct 17;16:9239. doi: 10.1038/s41467-025-64273-6 (PMC12534483; doi:10.1038/s41467-025-64273-6)
Supplement: Supplementary file 1 — Supplementary Information [file 41467_2025_64273_MOESM1_ESM.pdf]

Supplementary Materials for

Generation, Transmission, and Conversion of Orbital Torque by an Antiferromagnetic Insulator

Shilei Ding*, Paul Noël, Gunasheel Kauwtilyaa Krishnaswamy, Davitti Niccolò, Giacomo Sala, Marzia Fantauzzi, Antonella Rossi, Pietro Gambardella*.

*Corresponding author. Email: shilei.ding@mat.ethz.ch, pietro.gambardella@mat.ethz.ch

S1. Surface roughness

The thin-film heterostructures were deposited at room temperature on a SiO_2/Si substrate. Atomic force microscopy (AFM) images, presented in Fig. S1, reveal a relatively low surface roughness for these samples, with root mean square (RMS) values of 0.42 nm for Cu*(7)/Co(2) and 0.40 nm for Cu*(7)/CoO(2)/Co(5).

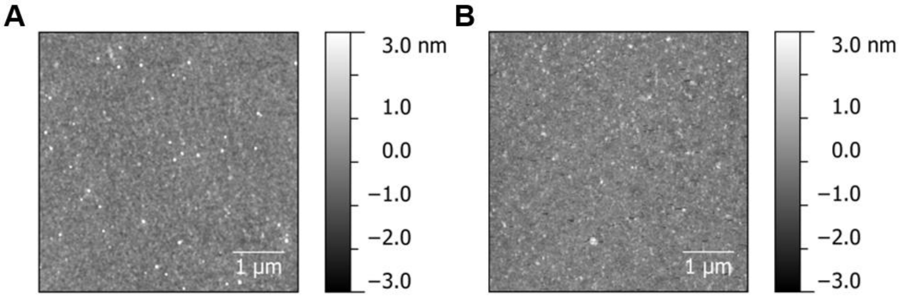

Fig. S1. Surface roughness. Topographic images of the surface of (**A**) Cu*(7)/Co(2) and (**B**) Cu*(7)/CoO(2)/Co(5) measured by atomic force microscopy. The RMS roughness is 0.42 nm and 0.40 nm, respectively.

S2. X-ray photoelectron spectroscopy

Survey and high resolution XP spectra were recorded at the beginning and after each sputtering cycle to monitor the surface composition of the samples as a function of depth. The XP-spectra were recorded and processed as described in the Materials and Methods section (main text).

Survey spectra (Fig. S2 **A**)

The surface of Cu*(7)/Co(5) shows the presence of Cu, C and O signals. The C and O signals are due to environmental contamination and oxidation since the sample was exposed to air before the XPS analysis. After 135 s of Ar^+ sputtering, the C and O signal vanish, and the Co signal appears. High-resolution spectra confirm these results (Fig. S2). After 255 s of Ar^+ sputtering both Co and Cu were detected and for prolonged sputtering time also the presence of Si peaks from the SiO_2 on Si wafer was observed. In Cu*(1.5)/Co(5), signals from Cu, C, and O are detected before the Ar^+ sputtering together with a small contribution of Co. Similarly, in Cu*(7)/CoO(2)/Co(5), Cu, C, and O are detected before sputtering, whereas Co peaks were observed only after 240 s of Ar^+ sputtering.

High-resolution spectra: chemical state analysis.

Copper (Fig. S2 **B**)

High-resolution spectra of Cu 2p and Cu $L_3M_{4,5}M_{4,5}$ were collected to determine the chemical state of copper at the surface and to monitor its composition as a function of depth. It is well known that the primary photoelectron line, Cu 2p3/2, has an identical binding energy for both Cu (0) and Cu (I), making it challenging to distinguish between these two oxidation states under typical laboratory conditions. Cu (II), however, is readily identified due to a distinct

chemical shift of the Cu $2p_{3/2}$ signal from elemental copper and the presence of shake-up satellites. Identification of Cu (I) in bulk materials is often achieved using X-ray excited Auger electron spectroscopy (XAES). In this study, Cu (II) was not detected. We analyzed the chemical state by resolving the five components attributed to Cu (0) and four associated with Cu (I) the Cu $L_3M_{4,5}M_{4,5}$ X-ray excited Auger spectra, using the curve-fitting procedure and quantification approach proposed in Ref. (1), which distinguishes and quantifies Cu (0) and Cu (I) species. In these spectra, copper shows main components at kinetic energies of 918.6 eV and 916.8 eV for Cu (0) and Cu (I), respectively, consistent with previous findings [2,3]. Before sputtering, copper was predominantly present as Cu (I) (Cu_2O) across all samples analyzed, particularly in the Cu*(1.5)/Co(5) sample, where Cu (I) was the only copper-bearing species detected. The binding energy values of each component and the curve-fitting parameters are provided in Table S1.

With increased sputtering time, the intensity of the component assigned to Cu (0) gradually rises, becoming predominant after approximately 120 seconds of Ar^+ sputtering across all samples. These measurements show that the natural oxidation of Cu in air leads to the formation of a Cu* layer with a thickness of about 2-3 nm, consistent with previous work[4].

Cobalt (Fig. S2 **C**)

Before sputtering, the Co $2p_{3/2}$ signal was detected only in sample Cu*(1.5)/Co(5) due to its thin copper layer (nominal thickness 1.5 nm), while for samples Cu*(7)/Co(5) and Cu*(7)/CoO(2)/Co(5), it became observable after approximately 120 seconds of Ar^+ ion sputtering. The Co signal in each case consists of two components, resolved by a curve-fitting procedure based on the analysis approach reported in Ref. (1). As per these references, the Co (0) and Co (II) signals display three and four components, respectively. Curve-fitting parameters are listed in Table S1. The binding energy for Co(0) (first peak of the multiplet: Co $2p_{3/2}$ (i) in Table S1) was 778.1 ± 0.1 eV, and CoO was found at 780.0 ± 0.1 eV (Table S1), in agreement with previous reports[1]. CoO was absent in Cu*(7)/Co(5), whereas in Cu*(1.5)/Co(5), its presence was attributed to oxygen diffusion through the thin Cu layer. In Cu*(7)/CoO(2)/Co(5), Cu $L_3M_{4,5}M_{4,5}$ spectra indicated metallic Cu after 240 seconds of Ar sputtering, while Co 2p spectra confirmed the presence of CoO, suggesting a Cu/oxide interface possibly contributing to the negative orbital polarization, as discussed in the main text.

Oxygen (Fig. S2 **D**)

The oxygen signal, O 1s, is also multicomponent. At the time 0 when the sample was not yet sputtered the components at 530.5 eV, 531.7 eV and at 532.7 eV are present and they are assigned to the presence of Cu_2O, CuOH and to adsorbed water (Table S1). The signal at 531.7 eV contains also the contribution to the carbon oxygen-containing functional groups present as surface contamination due to the exposure of the sample to the environment. The signal is modified upon sputtering time and by the end, the oxygen component at 532.9 eV assigned to SiO_2 is the only one detectable. No preferential sputtering effects are taken into account. A closer look at the shape of the oxygen signals during the compositional depth profile allowed us to identify the component at 529.6 eV, which is assigned to CoO, and a component at high binding energy (531.7 eV), which might be due to surface defect species that originate from the damage induced by Ar sputtering. This component was also reported by other authors[2].

Table S1: Curve fitting parameters, binding energy (BE) of the main photoemission lines, and kinetic energy (KE) of the components of the Cu $L_3M_{4,5}M_{4,5}$ Auger line. GL(x) line shape is a mixed Gaussian and Lorentzian product functions where x il the percentage of Lorentzian; GL(x)T(y) line shape describes a Gaussian/Lorentzian product function modified by an exponential factor (T) to take into account peak asymmetry due to multielectronic effects.The area ratio between the first peak of Co $2p_{3/2}$ and the other peaks of the multiplet splitting structure and between the first peak of Cu $L_3M_{4,5}M_{4,5}$ ascribed to both Cu and Cu_2O and the other components assigned to the two different chemical states was kept constant during the curve fitting; the area constraints are reported in the last column of the table.

Photoemission lines				
Signal	BE (eV)	Line Shape	FWHM	Area constraint
O 1s – Cu_2O	530.5 (0.1)	GL(30)	1.6 (0.1)	
O 1s – CuOH and contamination	531.7 (0.2)	GL(30)	1.6 (0.1)	
O 1s – water	532.5 (0.1)	GL(30)	1.6 (0.1)	
O 1s – CoO (i)	529.6 (0.1)	GL(30)	1.6 (0.1)	
O 1s – CoO (ii)	531.7 (0.2)	GL(30)	1.6 (0.1)	
O 1s SiO_2	532.9 (0.1)	GL(30)	1.6 (0.1)	
Co $2p_{3/2}$ Co – met (i)	778.1 (0.2)	GL(70)T(1)	1.0 (0.1)	
Co $2p_{3/2}$ Co – met (ii)	771.1 (0.2)	GL(30)	4.7 (0.1)	Co – met (i) x 0.11
Co $2p_{3/2}$ Co – met (iii)	783.1 (0.2)	GL(30)	4.7 (0.1)	Co – met (i) x 0.08
Co $2p_{3/2}$ CoO (i)	780.0 (0.2)	GL(30)	3.4 (0.1)	
Co $2p_{3/2}$ CoO (ii)	782.1 (0.2)	GL(30)	3.9 (0.1)	CoO (i) x 0.26
Co $2p_{3/2}$ CoO (iii)	785.5 (0.2)	GL(30)	3.7 (0.1)	CoO (i) x 0.02
Co $2p_{3/2}$ CoO (iv)	786.5 (0.2)	GL(30)	5.1 (0.1)	CoO (i) x 0.26
Cu $2p_{3/2}$ – Cu met and/or Cu_2O	932.6 (0.1)	GL(70)T(1.5)	1.7 (0.1)	
Cu $L_3M_{4,5}M_{4,5}$ Auger line				
Signal	KE (eV)	Line Shape	FWHM	Area constraint
Cu – met (i)	918.6 (0.2)	GL(80)	1.6 (0.1)	
Cu – met (ii)	916.5 (0.2)	GL(30)	1.9 (0.1)	Cu – met (i) x 0.3
Cu – met (iii)	913.5 (0.2)	GL(30)	2.3 (0.1)	Cu – met (i) x 0.2
Cu – met (iv)	919.2 (0.2)	GL(30)	1.1 (0.1)	Cu – met (i) x 0.1
Cu – met (v)	920.8 (0.2)	GL(30)	1.7 (0.1)	Cu – met (i) x 0.3
Cu_2O (i)	916.8 (0.2)	GL(70)	2.0 (0.1)	
Cu_2O (ii)	913.7 (0.2)	GL(30)	5.2 (0.1)	Cu_2O (i) x 0.6
Cu_2O (iii)	919.2 (0.2)	GL(30)	2.3 (0.1)	Cu_2O (i) x 0.4
Cu_2O (iv)	921.8 (0.2)	GL(30)	2.4 (0.1)	Cu_2O (i) x 0.1

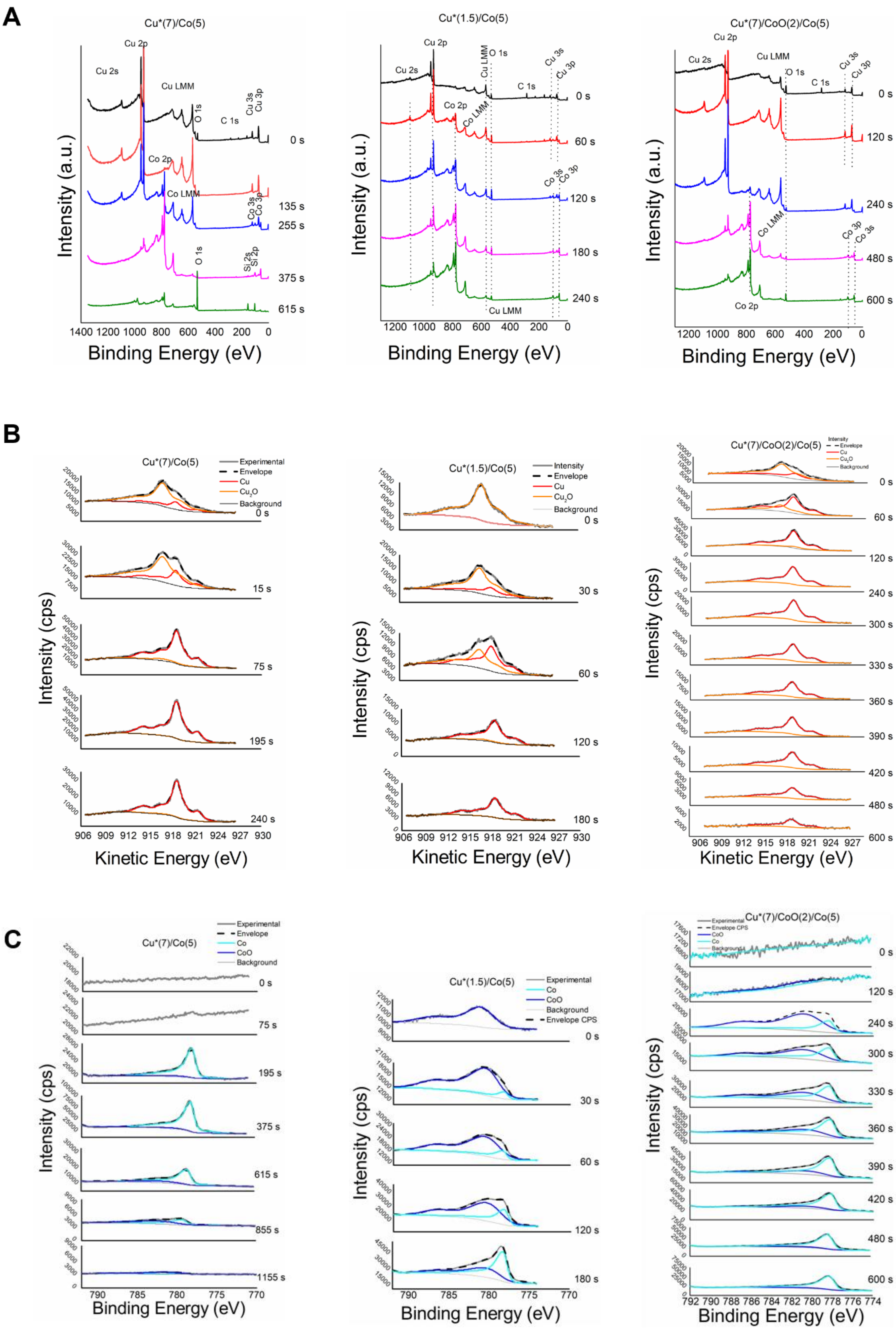

A
Cu*(7)/Co(5)
Cu 2s
Cu 2p
Cu LMM
O 1s
C 1s
Cu 3s
Cu 3p
Co 2p
Co LMM
Co 3s
Co 3p
Si 2s
Si 2p
0 s
135 s
255 s
375 s
615 s
Intensity (a.u.)
Binding Energy (eV)
1400 1200 1000 800 600 400 200 0
Cu*(1.5)/Co(5)
Cu LMM
60 s
120 s
180 s
240 s
1200 1000 800 600 400 200 0
Cu*(7)/CoO(2)/Co(5)
120 s
240 s
480 s
600 s
B
Experimental
Envelope
Cu
Cu2O
Background
15 s
75 s
195 s
240 s
Intensity (cps)
906 909 912 915 918 921 924 927 930
Kinetic Energy (eV)
Intensity
30 s
300 s
330 s
360 s
390 s
420 s
906 909 912 915 918 921 924 927
C
Co
CoO
855 s
1155 s
790 785 780 775 770
Binding Energy (eV)
Envelope CPS
792 790 788 786 784 782 780 778 776 774

D

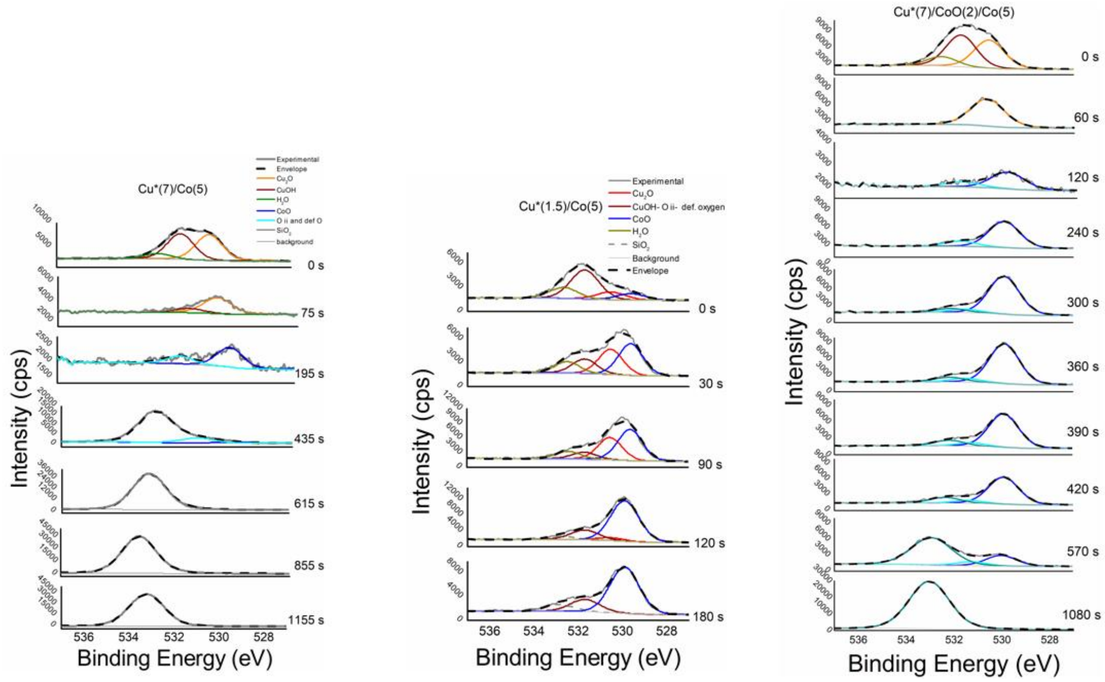

Fig. S2. X-ray photoelectron spectra. (**A**) XPS survey spectra obtained on samples Cu*(7)/Co(5), Cu*(1.5)/Co(5) and Cu*(7)/CoO(2)/Co(5) for different sputtering times. (**B**) Cu $L_3M_{4,5}M_{4,5}$ spectra indicating the presence of CuO_x at the surface of Cu*. (**C**) Co $2p_{3/2}$ spectra indicates the presence of CoO in Cu*(1.5)/Co(5) and Cu*(7)/CoO(2)/Co(5). (**D**) O 1s spectra.

S3. Electrical resistance of Cu*($t_{\mathrm{Cu*}}$)/Co(2) and Cu*/CoO(t_{CoO})/Co(2)

In the main text, we denoted naturally oxidized copper as Cu* and refer to $t_{\mathrm{Cu*}}$ as the total thickness of the Cu layer prior to oxidation. According to both XPS analysis and resistivity measurements, Cu* layers thinner than about 2 nm are fully oxidized, whereas thicker Cu* layers consist of a top oxide layer and a bottom Cu metal layer with a gradual transition between the two regions (Fig. 1 in the main text). The four-point longitudinal resistance of the Cu*($t_{\mathrm{Cu*}}$)/Co(2) samples, as a function of $t_{\mathrm{Cu*}}$ is shown in Fig. S3 **A**.

To further assess whether the CoO thin film is insulating, four-point resistance measurements were performed on 2-nm-thick and 27-nm-thick single-layer CoO samples. In both cases, the resistance exceeded the multimeter's measurement limit (200 MΩ), confirming that our sputtered CoO layers exhibit excellent insulating properties ($\rho > 182\ \Omega$ cm).

We compared the longitudinal resistance of Cu*(7)/CoO(t_{CoO})/Co(3) in samples with different CoO thickness. As shown in Fig. S3 **B**, we observe no significant change in resistance as the CoO thickness varies between 1 and 10 nm, indicating that the resistance of the CoO layer is significantly larger than that of the metallic Cu and Co layers for all samples investigated in this work.

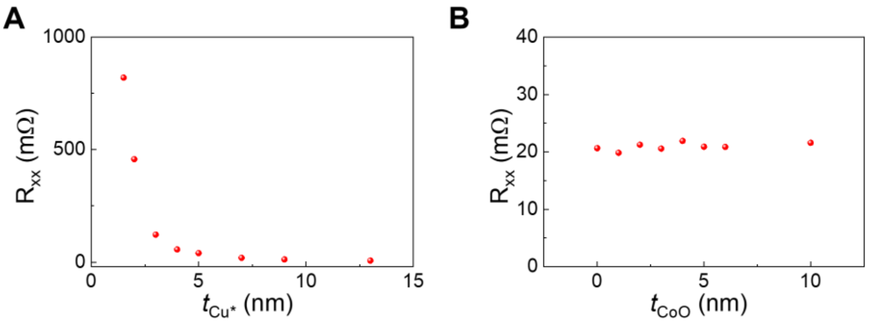

Fig. S3. Longitudinal resistance. (**A**). Cu* thickness dependence of the longitudinal resistance in Cu*($t_{\mathrm{Cu*}}$)/Co(2). (**B**) Dependence of longitudinal resistance in Cu*(7)/CoO(t_{CoO})/Co(3) as a function of CoO thickness.

S4. Anomalous Hall resistance and effective field $B_{\mathrm{dem+ani}}$

Figure S4 **A** displays the anomalous Hall resistance of Cu*(5)/Co(2) measured after photolithography and lift-off. The field at which the anomalous Hall resistance saturates corresponds to the effective demagnetization and anisotropy field $B_{\mathrm{dem+ani}}$ [5], which we estimate to be $B_{\mathrm{dem+ani}} = 1.5 \pm 0.1$ T with $R_{\mathrm{AHE}} = 38 \pm 1$ mΩ. For the Cu*(7)/CoO(2)/Co(3) sample, we meausre $B_{\mathrm{dem+ani}} = 1.5 \pm 0.1$ T with $R_{\mathrm{AHE}} = 12.8 \pm 0.2$ mΩ. Smaller value of R_{AHE} is due to the pronounced current shunting through Cu.

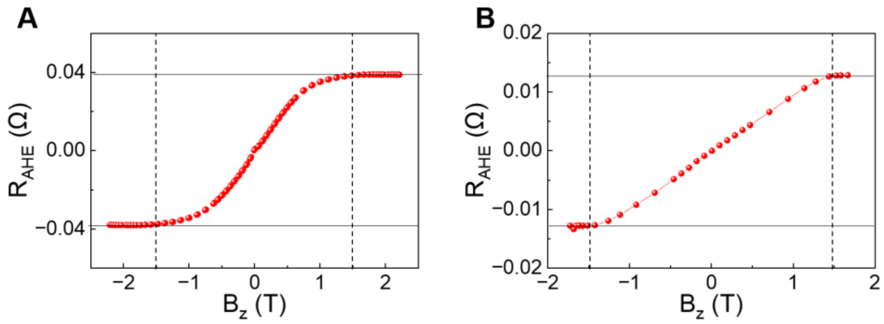

Fig. S4. Anomalous Hall resistance and effective field $\mathbf{B_{dem+ani}}$. (**A**) and (**B**): Anomalous Hall resistance as a function of the out-of-plane field for Cu*(5)/Co(2) and Cu*(7)/CoO(2)/Co(3) at 300 K, respectively. The applied ac current is 1 mA (peak value).

S5. Areal magnetization of Cu*($t_{\mathrm{Cu*}}$)/Co(2)

To quantify the extent of Co oxidation in thinner Cu* samples, we conducted magnetic hysteresis measurements. Based on changes in areal saturation magnetization, we estimated the Co and CoO layer thicknesses in Cu*($t_{\mathrm{Cu*}}$)/Co(2) samples, as illustrated in Fig. S5 **A**. Here, the areal saturation magnetization ($M_{\mathrm{S}} t_{\mathrm{Co}}$, where M_{S} represents the saturation magnetization and t_{Co} the thickness of the magnetic layer) increases with Cu* thickness, reaching a plateau, suggesting that a 3-nm-thick Cu* layer effectively prevents further oxidation of the Co layer beneath. Figure S5 **B** shows the hysteresis loops of Cu*(1.5)/Co(2), Cu*(5)/Co(2), and Cu*(7)/Co(2) samples at 300 K.

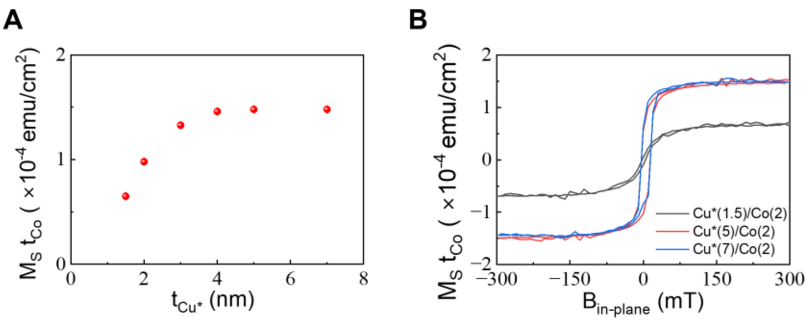

Fig. S5. Areal magnetization. (**A**) Areal saturation magnetization of the Cu*(t_{Cu*})/Co(2) samples. The decrease of the magnetization for the thinner Cu* layer indicates the formation of the CoO layer. (**B**) In-plane hysteresis loop for Cu*(1.5)/Co(2), Cu*(5)/Co(2), and Cu*(7)/Co(2) samples obtained at 300 K by SQUID.

S6. Current-induced torque in Pt/Co

We report measurements of the spin-orbit torques in Pt(4)/Co(3)/SiO_2(300nm)/Si as a standard sample for comparison of the torque efficiency and sign. The measurement shows a positive torque (Fig. S6), as expected for the positive spin Hall effect of Pt. The torque efficiency per unit applied electric field is $\xi_{DL} = \frac{2e}{\hbar E} M_S t_{Co} B_{DL} = 2.33 \pm 0.02 \times 10^5\ \Omega^{-1}\mathrm{m}^{-1}$. Here $M_S t_{Co} = 2.5 \times 10^{-4}$ emu/cm^2, and the longitudinal resistance of the sample is 78 Ω.

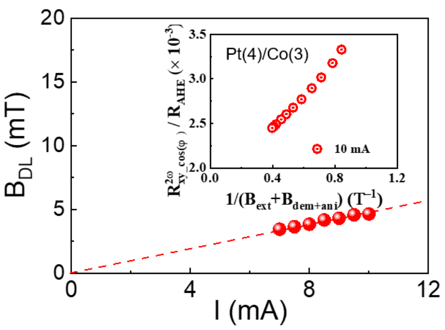

Fig. S6. Current-induced spin-orbit torque in Pt/Co. Current dependence of B_{DL} in Pt(4)/Co(3) measured at room temperature. Inset: cos φ component of $R_{xy}^{2\omega}$ (denoted as $R_{xy\_\cos\varphi}^{2\omega}$) plotted against $1/(B_{ext} + B_{dem+ani})$.

S7. Exchange bias in Cu*(t_{Cu*})/Co(2) full films

CoO/Co bilayers are a well-established model system for studying exchange bias effects[6-8]. Partial oxidation of Co in thin Cu*(t_{Cu*})/Co(2) layers can thus give rise to exchange bias. We characterized the exchange bias by measuring temperature-dependent hysteresis loops using SQUID magnetometry after cooling the samples in a magnetic field of 0.3 T from room temperature to the temperature of interest. We define B_+ and B_- as the positive and negative coercive fields, respectively, the exchange bias field $B_{EB} = \frac{1}{2}(B_+ + B_-)$, and the coercivity

$B_C = \frac{1}{2}(B_+ - B_-)$. Figure S7 shows the temperature dependence of B_{EB} and B_C for samples Cu*(1.5)/Co(2), Cu*(2)/Co(2), Cu*(3)/Co(2), and Cu*(7)/Co(2). The blocking temperatures, which are typically slightly below the Néel temperature[6], are approximately 150 K for Cu*(1.5)/Co(2) and Cu*(2)/Co(2). These samples also exhibit an enhanced coercive field, as typical for exchange-biased systems[6-8]. For thicker Cu* layers, oxygen diffusion is limited, preventing Co oxidation and thereby suppressing exchange bias. It is also noted that while Cu*(3)/Co(2) has a lower areal saturation magnetization than Cu*(4)/Co(2) (see Fig. S5 **A**), no significant exchange bias effect is observed in Cu*(3)/Co(2). This is attributed to the small and discontinuous oxidation of the Co layer protected by Cu*(3).

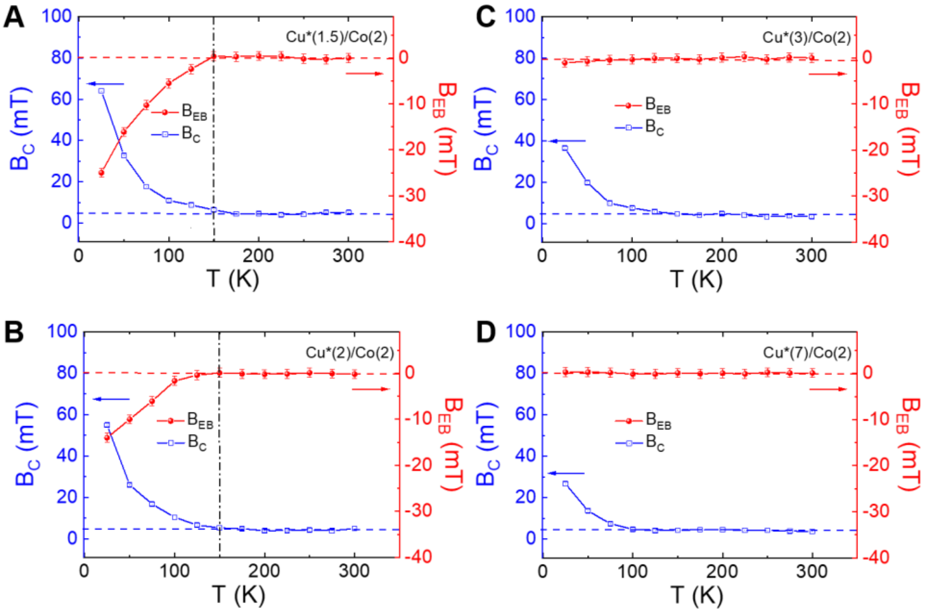

Fig. S7. Exchange bias effect in Cu*(t_{Cu*})/Co(2). Temperature dependence of the exchange bias field and coercive field in Cu*(1.5)/Co(2) (**A**), Cu*(2)/Co(2) (**B**), Cu*(3)/Co(2) (**C**), and Cu*(7)/Co(2) (**D**). The samples with thinner Cu* layers, Cu*(1.5)/Co(2) and Cu*(2/Co(2), show a finite exchange bias, consistent with the formation of an antiferromagnetic CoO layer. The blocking temperature in these samples is determined to be about 150 K from the onset of exchange bias (dashed lines).

S8. Magnetic properties of Cu*(7)/CoO(2)/Co(t_{Co}) full films

Cobalt oxidizes readily, and CoO/Co bilayers can be formed by introducing oxygen into the sputtering chamber after the deposition of Co . In this work, a mixture of 100 sccm Ar and 4 sccm O_2 was introduced into the chamber for 10 minutes at 4.3×10^{-3} mbar pressure to oxidize the Co surface. Subsequently, a Cu layer was deposited. For the Cu*(7)/CoO/Co sample obtained in this way, starting from a 5-nm-thick Co layer, we observe that the areal magnetization, $M_S t_{Co}$ is about 57% of that in the reference sample SiN(6)/Cu(7)/Co(5) (Fig.

S8 **A**). The $M_S t_{Co}$ values for Cu*(7)/CoO/Co samples with 5 and 7 nm Co layers are similar, suggesting that the CoO thickness obtained by post-deposition oxidation of Co is approximately 2 nm. In the main manuscript, we therefore label this sample as Cu*(7)/CoO(2)/Co(3). The CoO layer in the control samples SiN(6)/Cu(7)/CoO(2)/Co(3) and SiN(6)/Cu(7)/CoO(2)/Co(3) was obtained in the same way.

To confirm the presence of CoO in the post-deposition oxidized samples, we performed hysteresis loop measurements after field cooling in a 0.3 T field. Figures S8 **B**-**E** display the exchange bias field and coercive field temperature dependence for samples Cu*(7)/CoO(2)/Co(t_{Co}) with t_{Co} = 2, 3, 4, and 5 nm. The blocking temperatures for these samples are approximately 200 K.

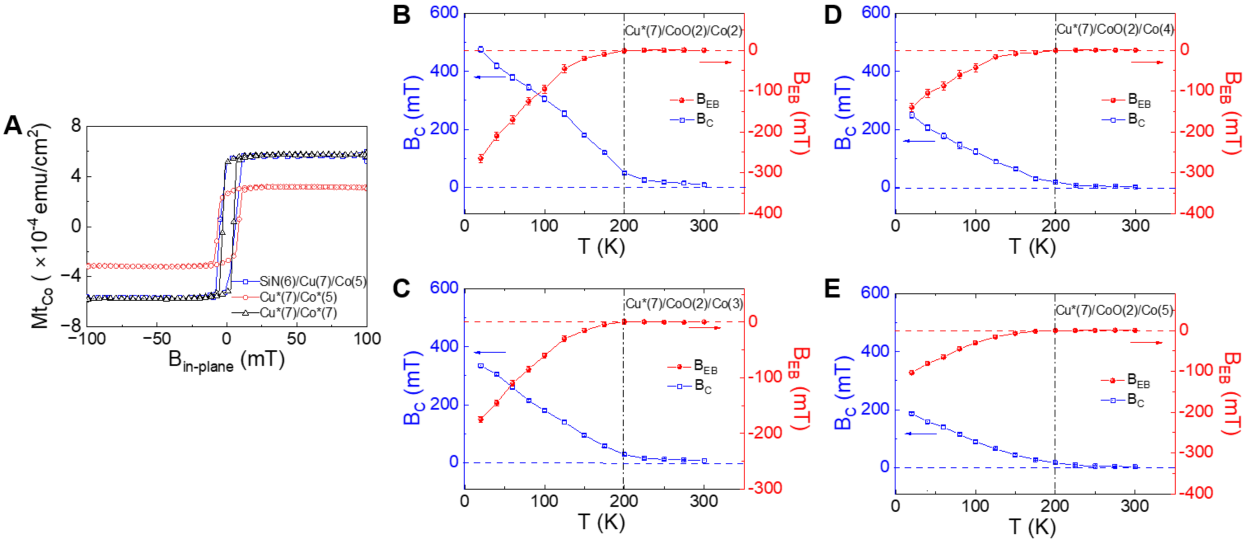

Fig. S8. Magnetic properties of Cu*(7)/CoO(2)/Co(t_{Co}). (**A**) In-plane hysteresis loop of the SiN(6)/Cu(7)/Co(5), Cu*(7)/CoO(2)/Co(3)), and Cu*(7)/CoO(2)/Co(5) samples obtained at 300 K by SQUID. Temperature dependence of the exchange bias field and coercive field of Cu*(7)/CoO(2)/Co(2) (**B**), Cu*(7)/CoO(2)/Co(3) (**C**), Cu*(7)/CoO(2)/Co(4) (**D**) and Cu*(7)/CoO(2)/Co(5) (**E**). The blocking temperatures are around 200 K, as indicated by the dashed lines.

S9. Temperature dependence of orbital torque in Cu*(1.5)/Co(2) and Cu*(2)/Co(2).

As established in Section S7, both Cu*(1.5)/Co(2) and Cu*(2)/Co(2) exhibit exchange bias, indicating CoO formation. Figures S9 **A** and **B** show the temperature dependence of orbital torque in these samples. A negative torque is observed, reaching a minimum magnitude near the blocking temperature, a trend also seen in Cu*(7)/CoO(2)/Co(3) (main text). Moreover, the orbital torque efficiency in Cu*(1.5)/Co(2) and Cu*(2)/Co(2) is lower than in Cu*(7)/CoO(2)/Co(3), likely due to reduced CuO_x formation at the Cu*/CoO interface, resulting in weaker orbital Rashba-Edelstein effect.

Upon reducing the temperature below T_B, the magnitude of the torque increases, consistently with the behavior observed in Cu*/CoO/Co trilayers. An explanation of the nonmonotonic temperature dependence of the orbital torque is provided in the main text. Additionally, we find that field cooling enhances the magnitude of the orbital torque in Cu*(1.5)/Co(2), similar to the Cu*(7)/CoO(2)/Co(3) result. Field cooling likely improves magnetic order at the CoO interfaces, enhancing the conversion and transmission of the orbital current.

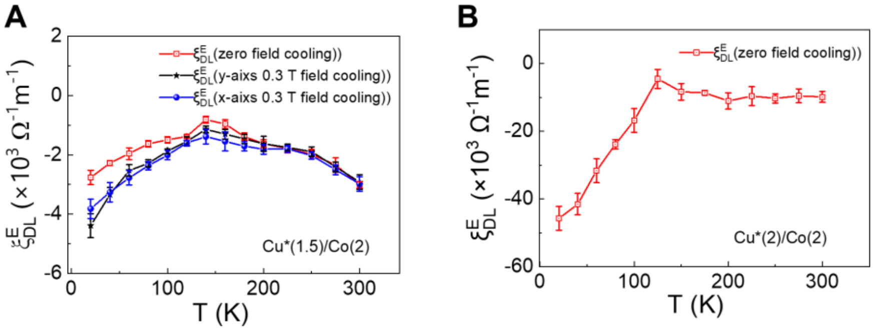

Fig. S9. Temperature dependence of the orbital torque efficiency in Cu*(1.5)/Co(2). (**A**) Temperature dependence of the orbital torque efficiency in Cu*(1.5)/Co(2), showing minimum magnitude close to the blocking temperature ($T_B \approx 150$ K). The orbital torque is enhanced by field cooling. (**B**) Temperature dependence of the orbital torque efficiency in Cu*(2)/Co(2).

S10. Temperature and thickness dependence of the orbital torque efficiency in a second batch of Cu*(7)/CoO(t_{CoO})/Co(3) samples

We conducted torque measurements on a second series of Cu*(7)/CoO(t_{CoO})/Co(3) samples to provide a consistent set of temperature and thickness-dependent data in samples grown under identical conditions. As shown in Figure S10 **A**, the orbital torque increases below T_B in all these samples, consistently with the measurements reported in the main text. The dependence of T_B on CoO thickness is presented in Figure S10 **B**. Additionally, the CoO thickness dependence of the orbital torque at 300 K and 50 K can be found in Figures S10 **C** and **D**, respectively. The decay constant of the torque efficiency in this new batch of samples is approximately 2.3 nm (3.4 nm) at 300 K (50 K).

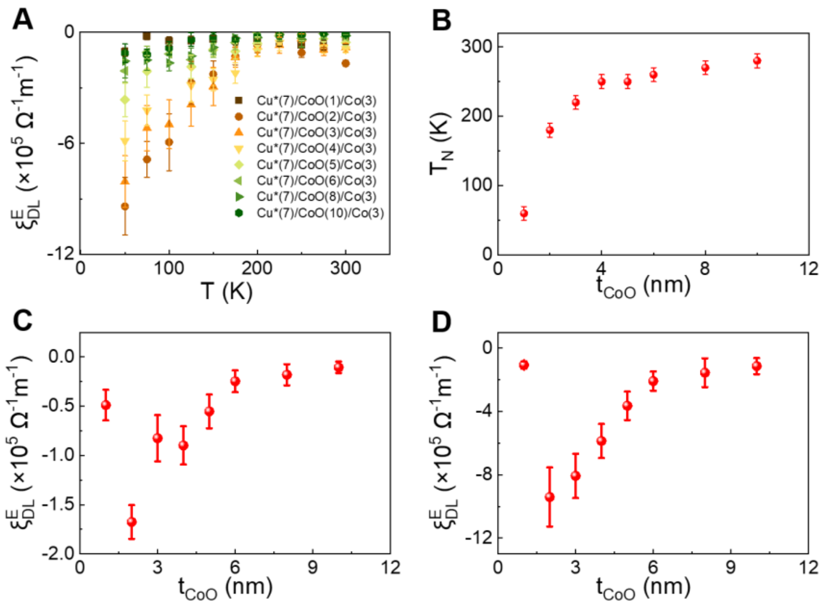

Fig. S10. Temperature and thickness dependence of the orbital torque in Cu*(7)/CoO(t_{CoO})/Co(3) (second batch). (**A**) Temperature dependence of the orbital torque efficiency. (**B**) CoO thickness dependence of the blocking temperature (T_B) from hysteresis loop measurements (see Section S7). (**C**) Orbital torque efficiency as a function of CoO thickness at 300 K (above T_B). (**D**) Orbital torque efficiency as a function of CoO thickness at 50 K (below T_B).

S11. Orbital Rashba-Edelstein magnetoresistance measurements on Cu*(7)/CoO(2)/Co(3)

We performed orbital Rashba-Edelstein magnetoresistance[9] measurements on Cu*(7)/CoO(2)/Co(3) at both 50 K and 300 K to search for signatures of spin-flop coupling in Cu*/CoO/Co, analogously to the spin Hall magnetoresistance measurements performed in Pt/NiO/CoFeB systems[10]. The magnetoresistance signal does not change significantly with temperature, indicating that spin flop coupling cannot explain the increase of the torque observed at low temperature.

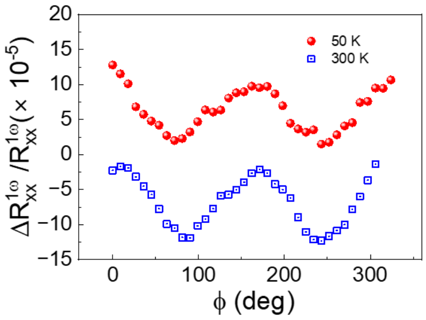

Fig. S11. Orbital Rashba-Edelstein magnetoresistance measurements of Cu*(7)/CoO(2)/Co(3) at 50 K and 300 K. The angle ϕ represents the orientation of the applied field relative to the current direction. The magnitude of the applied field is 0.94 T. The field is not large enough to saturate the magnetization of the Co layer, thus the curve slightly deviates from the usual $\cos^2(\phi)$ dependence.

S12. Temperature dependence of the orbital torque in Cu*(7)/MnO(2)/Co(3)

Figure S12 **A** shows the exchange bias field and coercivity as a function of temperature of Cu*(7)/MnO(t)/Co(3), from which we obtain $T_B = 60$ K. Figure S12 **B** presents the comparison of the temperature dependence of the orbital torque efficiency in Cu*(7)/MnO(2)/Co(3) and Cu*(7)/CoO(2)/Co(3) (second batch).

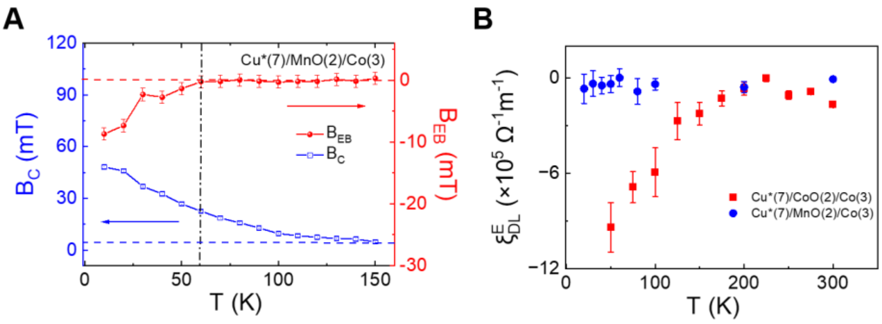

Fig. S12. Temperature dependence of orbital torque in Cu*(7)/MnO(2)/Co(3). (A) Temperature dependence of the exchange bias field and coercive field of Cu*(7)/MnO(2)/Co(2) (**B**) Comparison of the temperature dependence of orbital torque efficiency in the second batch of Cu*(7)/CoO(2)/Co(3) and Cu*(7)/MnO(2)/Co(3) samples.

S13.Exchange bias in Hall devices with training effect.

The coercive fields and exchange bias fields measured in the Hall bar devices after several training cycles are significantly smaller than those reported in Fig. 4 of the main text, which were measured by SQUID directly after field-cooling. For CoO/Co, a reduction of exchange bias by as much as 80% has been reported in literature after training[11]. In the Hall bar devices, we can estimate B_{EB} after training from the anisotropic magnetoresistance (AMR)[12]:

$$R_{\mathrm{xx}}^{1\omega} = R_0 + R_{AMR}\left(1 - \frac{\sin^2(\varphi-\varphi_s)}{1+\lambda^2+2\lambda\cos(\varphi-\varphi_s)}\right), \quad \text{(S1)}$$

where R_0 is a constant, R_{AMR} represents the AMR, $\lambda = \frac{B_{\mathrm{EB}}}{B_{\mathrm{ext}}}$, φ the angle between the applied field and the zero angle direction (alignment with the current axis), and φ_s is an unintentional misalignment of the zero angle direction with respect to current axis (*x*-axis).

Figure S13 **A** shows the fit of $R_{\mathrm{xx}}^{1\omega}$ for Cu*(7)/CoO(2)/Co(3) at a temperature of 20 K and $B_{\mathrm{ext}} = 600$ mT. By measuring the AMR for different values of B_{ext}, the exchange bias field can be determined from a linear fit of λ as a function of $\frac{1}{B_{\mathrm{ext}}}$, as shown in Fig. S13 **B**. In Fig. S13 **C**, the different values of B_{EB} are shown as a function of temperature. The blocking temperature is approximately 200 K, which aligns with the value obtained from the full film. This is expected since the Hall bar device samples were deposited simultaneously with the full film, followed by lift-off. The difference in exchange bias fields is between full films and Hall bars is attributed to the geometrical confinement and the training effect[11].

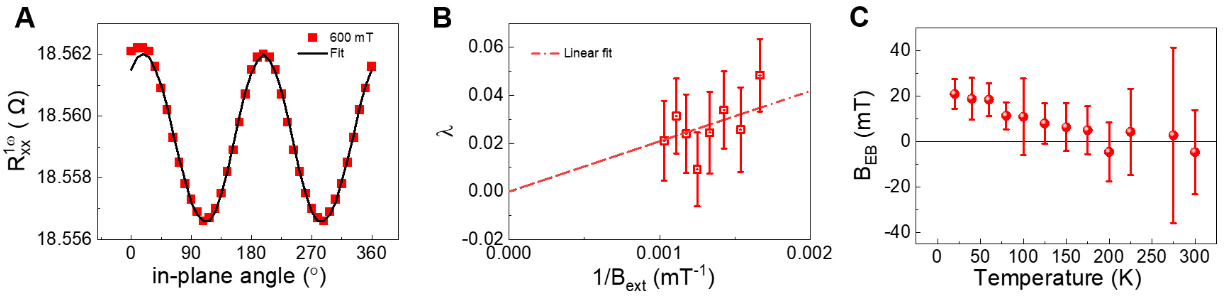

Fig. S13. Exchange bias of Cu*(7)/CoO(2)/Co(3). (**A**) In-plane angle dependence of $R^{1\omega}_{xx}$ at 20 K the solid curve is the fit with Eq. (S1). (**B**) λ as a function of $\frac{1}{B_{ext}}$, the slope indicates the exchange bias field B_{EB}. (**C**) Temperature dependence of exchange bias field B_{EB}.

Reference

1. Petitto, S. C., Marsh, E. M., Carson, G. A. & Langell, M. A. Cobalt oxide surface chemistry: The interaction of CoO(100), Co_3O_4(110) and Co_3O_4(111) with oxygen and water. *J. Mol. Catal. A Chem.* **281**, 49–58 (2008).
2. Biesinger, M. C. et al. Resolving surface chemical states in XPS analysis of first row transition metals, oxides and hydroxides: Cr, Mn, Fe, Co and Ni. *Appl. Surf. Sci.* **257**, 2717–2730 (2011).
3. Cocco, F. et al. Nanosized surface films on brass alloys by XPS and XAES. *RSC Adv*. **6**, 31277–31289 (2016).
4. Ding, S. et al. Observation of the Orbital Rashba-Edelstein Magnetoresistance. *Phys. Rev. Lett*. **128**, 67201 (2022).
5. Avci, C. O. et al. Interplay of spin-orbit torque and thermoelectric effects in ferromagnet/normal-metal bilayers. *Phys. Rev. B* **90**, 224427 (2014).
6. van der Zaag, P. J. et al. Difference between Blocking and Néel Temperatures in the Exchange Biased Fe_3O_4/CoO System. *Phys. Rev. Lett*. **84**, 6102–6105 (2000).
7. Tang, Y. J., Smith, D. J., Zink, B. L., Hellman, F. & Berkowitz, A. E. Finite size effects on the moment and ordering temperature in antiferromagnetic CoO layers. *Phys. Rev. B* **67**, 54408 (2003).
8. Gruyters, M. & Riegel, D. Strong exchange bias by a single layer of independent antiferromagnetic grains: The CoO/Co model system. *Phys. Rev. B* **63**, 52401 (2000).
9. Ding, S. et al. Observation of the Orbital Rashba-Edelstein Magnetoresistance. *Phys. Rev. Lett*. **128**, 67201 (2022).
10. Zhu, D. et al. Sign Change of Spin-Orbit Torque in Pt/NiO/CoFeB Structures. *Phys. Rev. Lett*. **128**, 217702 (2022).
11. Polisetty, S., Sahoo, S. & Binek, C. Scaling behavior of the exchange-bias training effect. *Phys. Rev. B* **76**, 184423 (2007).
12. Gong, J. et al. Exchange bias effect modified asymmetric magnetization reversal in Ni/$YMnO_3$ multiferroic bilayers. *Appl. Surf. Sci.* **368**, 44–48 (2016).
